# Dr. H. G. P. Spencer

**Published:** 1899-09

**Authors:** 


### OBITUARY.

Dr. H. G. P. Spencer, of Watertown, who for more than half a century had been in the successful practice of medicine in that city, died June 27, 1899, at Minneapolis, Minn., where he had been spending sometime in an attempt to recuperate his health.

Dr. Spencer was one of the principal founders of the reorganised Jefferson County Medical Society in 1868. He was the first president of the society, and was its delegate to the state medical society and censor in 1872, 1873 and 1883. In 1883 he was vice-president of the Medical Society of the State of New York. Dr. Spencer has enjoyed a large and lucrative private and consultation practice, and was a bold and successful surgeon until advancing years and failing sight compelled him to relinquish this branch of his profession.

He was the president of the City Medical Society at the time of his death, having been unanimously elected to that office at the last annual meeting in January.

Besides his wife, Dr. Spencer is survived by two sons, Dr. James D. Spencer and Dr. Gordon P. Spencer, of Watertown; two daughters, Mrs. Ella Martyn, of New York, and Mrs. E. H. Myers, of Carthage, and one brother, Dr. Gustavus N. Spencer, of Philadelphia.

At a special meeting of the Jefferson County Medical Society, at the office of Dr. J. M. Crawe, Jr., for the purpose of attending the funeral of Dr. Spencer, the following members were present: President, Dr. J. M. Crawe; vice-president, G. N. Cannon; secretary, C. M. Bibbins; Drs. Charles Kimball, Hoyt, Ives, George Foote, of Dexter; H. H. Wood, Calkins, J. M. Crawe, Sr., Rodenhurst, Philadelphia; O. C. Eastman, Willard, Gregor, Mason, Cape Vincent; A. B. Stevens, Goodale, Deane, Rexford, Kellow, Brown, Frank Massy, Brownville; Dick, McCaw, Geo. Sylvester, Black River; Joslin, M. L. Smith, Monroe Smith and John Jones, Evans Mills.

Dr. J. M. Crawe, Sr., chairman of committee, read the following resolutions, which on motion were adopted:

WHEREAS, Death has removed from amongst us one of our oldest and most widely known members and the first president of this society at its reorganisation in 1868; therefore, be it

*Resolved*, That, while yielding to the inevitable, we individually and collectively and as a society, recognise that in the death of Dr. H. G. P. Spencer we have lost a friend and the community at large an honored citizen.

*Resolved*, That as physicians, we knew him as one to whom we could turn in time of need for counsel and advice. He never failed to respond, drawing from his store of knowledge, gained by years of study and experience in the sick room, by the bedside of the sick and the suffering, be they rich or poor.

*Resolved*, That, deeply sensible of our loss, we wish to express our heartfelt sorrow and regret at his sudden passing from amongst us.

*Resolved*, That to his family, and especially to his wife, we desire to extend our deepest sympathy in this, their irreparable loss, praying the Father above may in His good time bind up their wounds.

*Resolved*, That these resolutions be published in all the city papers; that an engrossed copy, bearing the seal of the society, be presented to the bereaved family, and a copy placed on the files of the society.

J. MORTIMER CRAWE, M. D.

A. W. GOODALE, M. D.

GILBERT CANNON, M. D.

